# Macrophages and endothelial cells in the neurovascular unit

**DOI:** 10.1007/s00401-024-02697-y

**Published:** 2024-02-12

**Authors:** Jan Wenzel, Markus Schwaninger

**Affiliations:** 1 Institute of Experimental and Clinical Pharmacology and Toxicology, Center of Brain, Behavior and Metabolism, University of Lübeck, Lübeck, Germany; 2 German Research Centre for Cardiovascular Research (DZHK), partner site Hamburg/Lübeck/Kiel, Lübeck, Germany

In the brain, different types of cells interact and integrate complexly, providing not only intrinsic neuronal circuits but also a platform for communication that regulates all body functions. There has been considerable work published in recent years on two of those cell types and their interaction with the periphery, namely endothelial cells and macrophages of the brain. Here, we introduce a cluster of articles reviewing recent findings and insights into macrophage and vascular cell functions in the brain. These reviews originate from members of the EU consortium ENTRAIN (ENdoThelial macRophage Alliance In Neuroinflammation), which combined the expertise of researchers in the field of the brain´s vasculature and immune system in 14 projects across Europe.

Historically, the borders of the central nervous system have sparked scientific debates, which have divided the neuroscience community. Current controversial issues are related to the outflow of brain fluids or the neurobiology of the meninges [1, 10, 12, 13]. There has been disagreement in the past regarding whether vessels are neuroscience topics. Traditionally, neurophysiologists would have seen vessels outside the central nervous system, and stroke patients in some countries were treated on internal medicine wards, not neurological wards. Against this background, in 2001 a group of experts convened by the National Institute of Neurological Disorders and Stroke, USA, propagated the concept of the neurovascular unit [9]. The term “neurovascular unit” has had a significant impact on science policy and in the scientific community as it highlights the common ground of diverse observations, namely that nerve cells and vessels work together. In a process called neurovascular coupling, vessels and parenchymal cells adapt perfusion to neuronal activity, which is the basis for functional magnetic resonance imaging [9]. As another example, the blood–brain barrier is formed by the interaction between parenchymal and endothelial cells [1, 8].

There is no doubt that the concept of the neurovascular unit has a strong integrative power but the devil is in the details. A challenging task in the last two decades has been defining the cellular constituents of the neurovascular unit and describing their interactions. The list of cells involved in the neurovascular unit started with neurons, astrocytes, pericytes, and endothelial cells [8] but has since expanded. Recently, it was suggested to include extra-neural cells [17]. This cluster review focuses on brain macrophages which have only recently been appreciated as a key building block of the unit. Brain macrophages are related to endothelial cells and closely interact with them. This is demonstrated by the fact that some endothelial cells derive from the same precursor cells as brain macrophages [14]. Both cell types are involved in the vascular response to CO_2_ [4, 18]. Moreover, both endothelial cells and perivascular macrophages (PvMΦ) mediate the detrimental effect of IL-17 on neurovascular function and cognition in a model of salt-sensitive hypertension [15]. Therefore, these two cell types contribute to vascular reactivity and brain health. Defining the role of brain macrophages and endothelial cells in the neurovascular unit has gained complexity with the advent of single-cell RNA sequencing and other techniques that have provided new insight into the diversity of these cell types. A current concept of the role of brain macrophages in the neurovascular unit should take the different developmental trajectories and functions of microglia, PvMΦ, or monocyte-derived macrophages into account. Likewise, endothelial cells are heterogeneous along the vascular tree with emerging implications for the neurovascular unit.

The article “Origin, function and fate of CNS-associated macrophages in health and disease—an update” by Adrian Dalmau and colleagues focuses on these cells and includes a comprehensive overview of current knowledge about macrophages in the brain and spinal cord [5]. Among different subpopulations of macrophages specialized according to localization and functionality, Dalmau and colleagues review the brain’s extra-parenchymal macrophage population, the so-called CNS-associated macrophages (CAMs), which differ from microglia in several ways. Interestingly, microglia and CAMs originate from erythromyeloid precursors during development. This is a cell population that also contributes to the formation of endothelial cells in the brain [14]. In that article, recent advances are discussed that have led to a more differentiated view of the CAM population and its development, for example, the finding that the specification of CAMs to PvMΦ occurs after birth and is supported by vascular cells [11]. The role of CAMs during cerebrovascular and neurodegenerative diseases is also addressed and contrasted with the brain infiltration of myeloid cells during neuroinflammatory diseases.

The interplay between macrophages and endothelial cells is the topic of the article “Endothelial cells and macrophages as allies in the healthy and diseased brain” by Adam Denes, Cathrin Hansen and colleagues [6]. A significant step forward in understanding the role of macrophages in vascular regulation is the finding of direct contacts between endothelial cells and microglia. This finding and its implications are discussed in detail. They provide the basis for the intriguing discovery that microglia modify brain perfusion and neurovascular unit function [2, 4]. An interaction between these two cell types also includes the maintenance of blood–brain barrier integrity which is primarily built up by endothelial cells but affected by macrophages as well, especially during diseased states. The authors explain the different modes of how macrophages influence the tightness of the barrier which plays a protective role mainly during already impaired states like mild hypoxia or old age. Interestingly, an impaired blood–brain barrier is an early indicator of cognitive dysfunction, too [16].

A final article entitled “β-amyloid, Tau and α-synuclein versus brain macrophages, endothelial cells and pericytes: the engineers of blood–brain barrier dysfunction in acute and chronic neurological disorders” by Ying-Chieh Wu, Tizibt Bogale and colleagues examines specific mechanisms of how cells of the neurovascular unit are affected by and affect neurodegenerative diseases [19]. The authors summarize evidence that the accumulation of the formerly-mentioned proteins in the form of fibrils is a common factor in diseases accompanied by blood–brain barrier disruption. Interestingly, macrophages and endothelial cells are involved in the clearance of these protein deposits which are a by-product of neuronal activity and can impair neurovascular function. In addition to Alzheimer’s and Parkinson’s diseases, the authors focus on cerebral amyloid angiopathy in which β-amyloid deposits can be found around vessels. In all these diseases, a lack of clearance of protein deposits might be causal for clinical progression. Besides phagocytotic activity, macrophages might support protein fibril clearance by affecting cerebrospinal flow rate [7]. On the other hand, endothelial cells express specific transporters involved in protein efflux out of the brain, preventing fibril deposition, too. The authors discuss how an impaired brain vasculature, including endothelial cells and pericytes, might contribute to disease onset and progression.

Endothelial cells and macrophages are involved in most brain diseases. Their role and interaction within the neurovascular unit provide common mechanisms that may underlie diverse disease processes. Despite the fact that the concept of the neurovascular unit is already seasoned, it is still a very relevant framework for neuropathology. In the future, additional factors and cells might be discovered to be part of the functional neurovascular unit, for example perivascular fibroblasts which have just been recently described to be a heterogenous and localization-dependent cell population in the brain different from mural cells [3, 13].
